# Histological characteristics of macrodontic cheek teeth of guinea pigs

**DOI:** 10.1186/s12917-023-03567-7

**Published:** 2023-01-19

**Authors:** Justyna Ignaszak-Dziech, Piotr Kuropka, Tomasz Piasecki

**Affiliations:** 1 Center for the Diagnosis and Treatment of Exotic Animals, Veterinary Clinic “Zwierzyniec”, Bulwar Ikara 31B, 54-130 Wroclaw, Poland; 2 Division of Histology and Embryology, Department of Biostructure and Animal Physiology, Wroclaw University of Environmental and Life Sciences, Ul. Norwida 25, 50-375 Wrocław, Poland; 3 Department of Epizootiology and Clinic of Bird and Exotic Animals, Wroclaw University of Environmental and Life Sciences, Pl. Grunwaldzki 45, 50-366 Wrocław, Poland

**Keywords:** Guinea pig, Macrodont, Odontoma, Hamartoma, Structural alteration, Cheek teeth

## Abstract

Macrodontia is the enlargement of tooth dimensions of different ethologies. This work aims to show a histological evaluation of macrodontic teeth in guinea pigs. The material was obtained from animals postmortem. Ninety structural changes derived from 24 guinea pigs were evaluated. All teeth used in the study showed macrodontic changes. The samples were decalcified, dehydrated and embedded in paraffin. Material was cut in the transverse and longitudinal planes in relation to the alveolar bone. Histological evaluation included apical bud cells, pulp cavity cells, periodontium, dentin, enamel, cementum and alveolar bone tissue. Individual elements were evaluated with respect to their morphology and distribution. Moreover, the arrangement of the individual hard tooth structures was assessed on the teeth. No atypia was found among the cells that make up the apical bud, pulp or periodontal cavity. Displacement of periodontal cells or odontoblasts towards the pulp cavity as well as disorganization of the cell system in the pulp cavity were observed. Changes in the dentine ligaments and the reconstruction of the alveolar bone were also observed in areas where dentine and cement systems were affected. Dental slides were observed and showed that the enamel is also involved in structural remodelling of the pulp cavity. The histological assessment revealed that structural changes in macrodontic teeth involve the rearrangement of typical tooth tissues.

## Introduction

Guinea pigs (Cavia porcellus) belong to the order Rodentia and suborder Hystricomorpha. The dentition of this species consists of 20 teeth in 4 arcades, and the dental formula is as follows:${\mathrm{I}}_{1}^{1}{\mathrm{C}}_{0}^{0}{\mathrm{P}}_{ 1}^{1}{\mathrm{M}}_{3}^{3}$. All teeth are (a) hypsodontic, i.e., with a long crown, which can be divided into clinical and reserve crown; (b) aradicular, i.e., the apex remains open throughout all life and does not create a root; and (c) elodontic, i.e., growing throughout the entire lifetime and constantly wearing down in the mouth cavity [1]. The histological study we conducted concerned only the cheek teeth of guinea pigs.

**Fig. 1:**
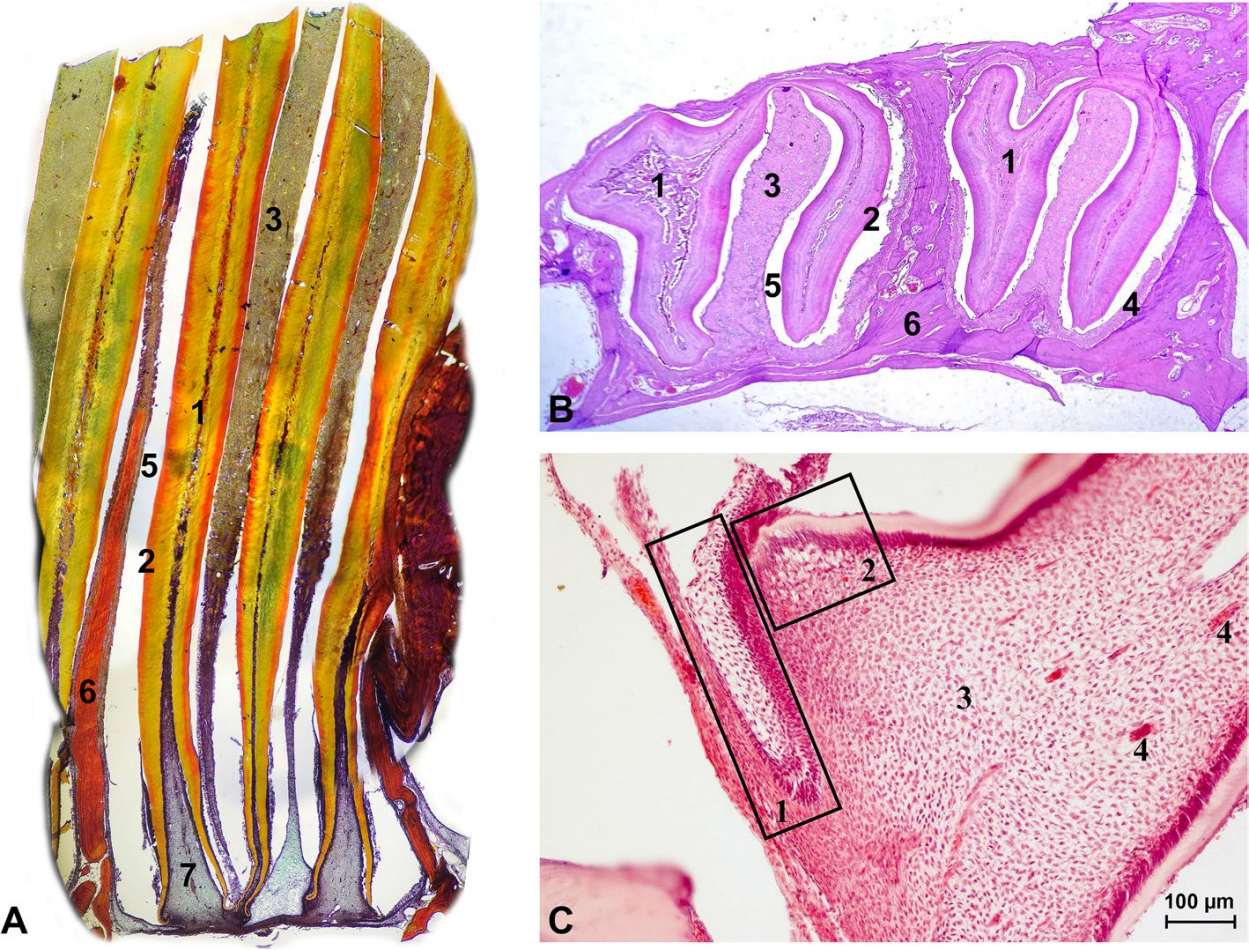
Histological image of a healthy guinea pig cheek tooth. 1a. Longitudinal section of a cheek tooth (van Gieson staining, × 10). 1b. Transverse section of M2 and M3 teeth (H&E, × 10). 1. Osteodentin, 2. dentin, 3. cartilage-like cementum, 4. periodontium, 5. enamel space, 6. alveolar bone, 7. pulp cavity. 1c. Longitudinal section of a cheek tooth apex (H&E, x100). 1. Zone of enamel production with stellate reticulum, 2. odontoblasts and predentin, 3. germinal tissue, 4. blood vessels

Constant growth of the cheek teeth of the guinea pig is possible due to the presence of the apical bud, within which stem cells capable of dividing are located. Cells of epithelial origin, which are responsible for the production of enamel, contact odontoblasts, which are responsible for the production of dentin. The space that is limited by the dentin is called the pulp cavity, and it is filled with connective tissue, cells of mesenchymal origin, vessels and nerves. On the longitudinal section of the cheek tooth, we can see two pulp cavities, which at the bottom of the alveolus join together to form the tooth apex. The space between the alveolar bone and the tooth is called the periodontal space, and it is filled with cells of mesenchymal and epithelial origin, vessels, nerves, and collagen fibres forming periodontal ligaments. The periodontal ligaments provide stabilization of the tooth in the alveolus as a result of the combination of the bone and the cementum pearls on the enamel surface. In addition to cementum pearls, we can distinguish the acellular cementum on the buccal surface of the maxillary cheek teeth and on the lingual surface of the mandibular cheek teeth as well as cartilage-like cementum between tooth pillars [2–6] (Fig. 1).

In this study, only teeth with structural alterations leading to enlargement of the tooth size, which is called macrodontia, were examined. Macrodontia is a term that appears rarely in the specialized literature [7–10], and it is interchangeably used with “giant tooth” or “crippled tooth” [7]. This paper aims to show the histological characteristics of structural changes in the macrodontic cheek teeth of guinea pigs.

## Materials and methods

The material was obtained during routine necropsies performed at the Department of Epizootiology and Clinic of Birds and Exotic Animals, Wroclaw University of Environmental and Life Science. Macroscopic evaluation of dental arcades was conducted. The dental arcades were collected from 24 animals (14 males and 10 females, aged 1.5 to 6.5 years, mean 5 years) in which at least one tooth showed structural changes on the occlusal surface or had structural alterations and was larger than the rest of the teeth from that arcade. A total of 72 structurally changed teeth were found in the 44 acquired dental arcades.

## Histological examination

Twenty-six dental arcades were selected for histological examination, and 47 teeth with morphological changes were found. The material was fixed in 10% buffered formalin for 72 h and then cleaned from soft tissues. The next step was to decalcify the material with ethylenediaminetetraacetic acid (EDTA) and hydrochloric acid in prep using a TBD – 1 Rapid Decalcifier (Thermo Scientific). After 36 h, the material was rinsed in tap water, dehydrated in ascending grades of alcohol, cleared in xylol and embedded in paraffin wax. Specimens of 5 µm thickness were made in the transverse and longitudinal plane in relation to the long axis of the alveolus. The specimens were stained with haematoxylin and eosin (H&E) [11].

The section slides were analysed and photographed using a Nikon Eclipse 80i microscope at magnifications of 40x, 100x, and 200x (Nikon, Melville, NY) provided with a video camera and NIS-Elements AR 2.30 (Nikon, Melville, NY) software. The evaluation included apical bud cells, pulp cavity cells, periodontium, dentin, cementum, and alveolar bone tissue. The tissues were evaluated regarding distribution and morphology.

## Histological examination using ground sections

Twenty-five morphologically changed teeth within 18 dental arcades were selected for the study. The material was fixed in 10% buffered formalin, and the fixed material was cleaned from soft tissues. Twelve morphologically changed teeth were isolated from dental arcades and cut into 3 equal parts perpendicular to the long axis of the alveolus using a sectioning machine with a diamond disc under water spray. The remaining 13 teeth with morphological changes were cut within the arcade, thus preserving the alveolar bone structure. The cutting of the material was performed in the same way as for the isolated teeth. The obtained fragments of the dental crowns and dental arcades were subject to abrasion with papers with gradually reduced granularity (500, 700) until transparent preparations were obtained. The thickness of the specimens was measured. Material with a thickness of approximately 100 µm was placed on clean glass slides. The section slides were analysed and photographed using a Nikon Eclipse 80i microscope at magnifications of 40x, 100x, and 200x (Nikon, Melville, NY) provided with a video camera and NIS-Elements AR 2.30 (Nikon, Melville, NY) software. The evaluation included distribution of the individual hard elements of the tooth and the alveolar bone [11].

## Results

### Distribution of the alterations

**Fig. 2:**
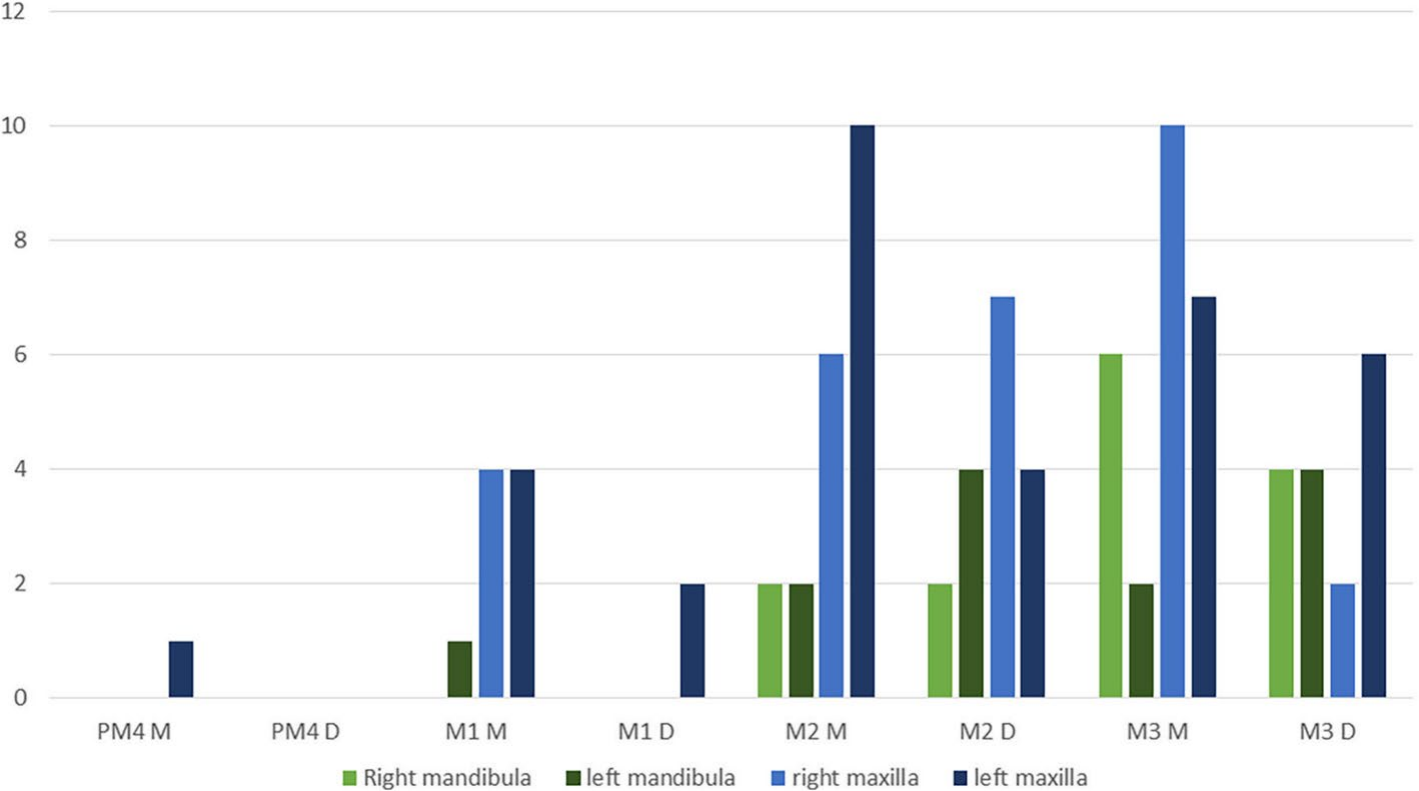
Distribution of the structural changes within macrodontic cheek teeth

A total of 72 altered teeth (PM4: 1, M1: 11, M2: 28, M3: 32) within 44 dental arcades were examined. First, the distribution of the alterations within the crown was evaluated. On the transverse section of the crown, the mesial (anterior) and distal (posterior) parts were determined in an “I” and a “V” shape, respectively. In 54/72 (75.0%) cases, structural change was found in only one part of the tooth crown (37 (68.52%) in the mesial part, 18 (31.48%) in the distal part). In 18/72 (25.0%) cases, structural changes were found in both the mesial and distal parts. In such situations, each alteration was evaluated individually in terms of morphology in further stages of work. A total of 90 structural changes were found within 72 macrodontic cheek teeth (Fig. 2).

**Fig. 3:**
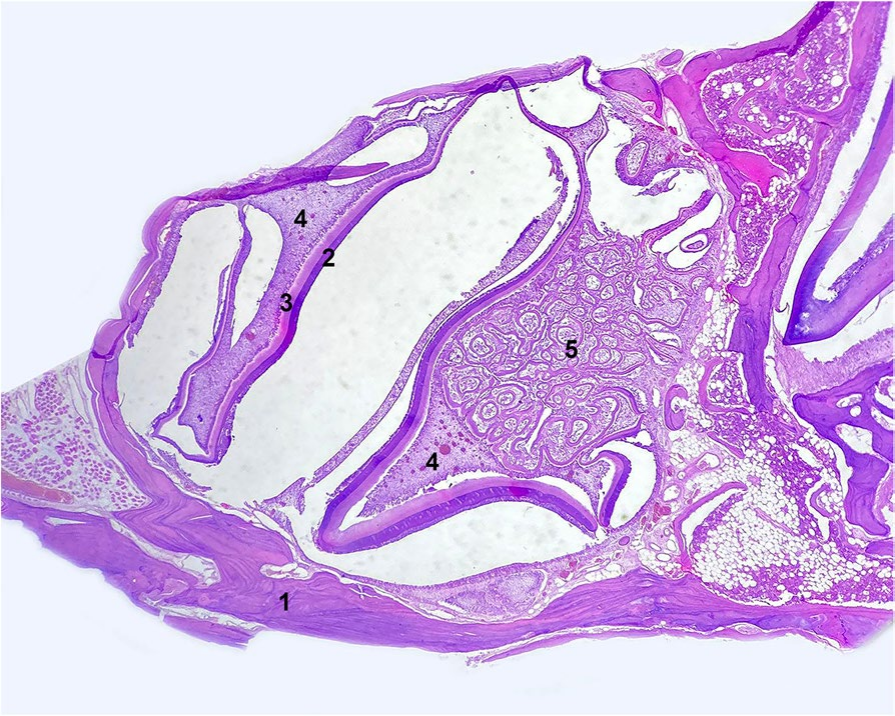
Transverse section of the M2 tooth of the guinea pig with structural alteration within the pulp cavity (H&E, × 10). 1. Alveolar bone, 2. enamel matrix, 3. dentin, 4. germinal tissue, 5. structural alteration built of tubular dentin, osteodentin and pulp cells

**Fig. 4:**
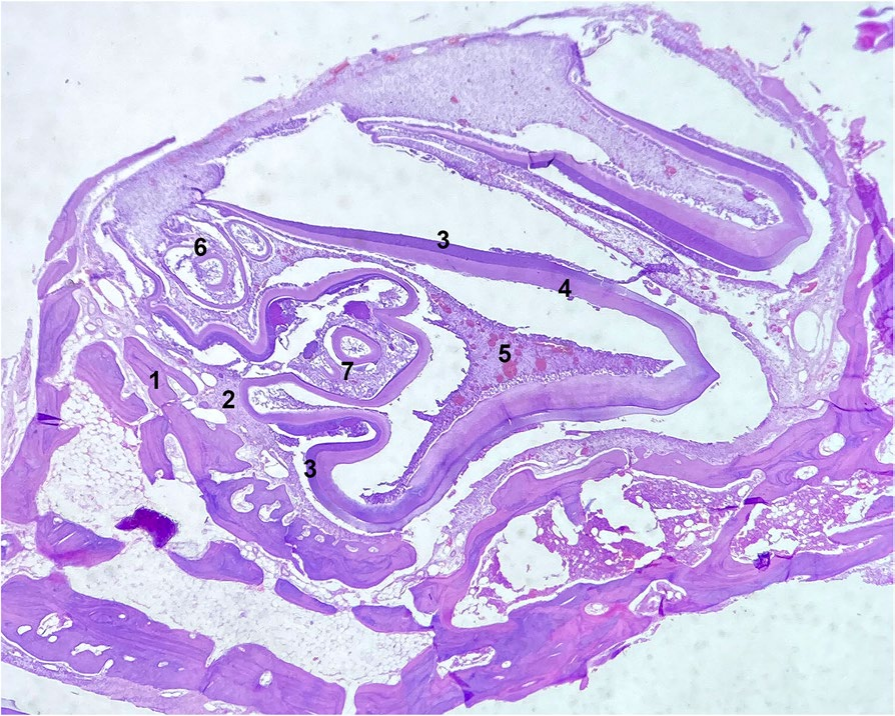
Transverse section of M2 tooth of a guinea pig with a structural alteration within the pulp cavity and deformation of the dentin (H&E, × 10). 1. Alveolar bone, 2. periodontium, 3. enamel matrix, 4. dentin, 5. germinal tissue, 6. structural alteration within the pulp cavity built of tubular dentin and pulp cells, 7. structural alteration with deformation of the dentin and protrusions into the pulp cavity built of tubular dentin, periodontium cells and cementum

During evaluation of the distribution of changes, it was observed that structural remodelling occurred within the pulp cavity or at the edge of the tooth crown. First, it was observed that changes in cell and tissue distribution occurred in the central part of the pulp cavity, whereas the dentin forming the walls of the pulp cavity was not subject to deformation (Fig. 3). Such changes were found in 45/90 (50%) cases. In 43/90 (47.78%) cases, various degrees of dentin deformation and dentin indentations (concavities) from the periodontal space towards tooth pulp were observed (Fig. 4). In such cases, no cell or tissue distribution changes within the pulp cavity were observed. In 2/90 (2,22%) cases, the entire crown of the tooth underwent structural remodelling, and it was not possible to determine whether the changes were derived from the pulp cavity or the periodontal space.

## Morphology of the alterations

No atypia of tooth building cells was found, only the rearrangement of the cells and tissue that were physiologically occurring within the tooth crown. The following tissues were observed to be involved in the construction of changes: osteodentin, tubular dentin, dental pulp cells, and cartilage-like cementum or the cells that are precursors of cartilage-like cementum. The data on the contribution of individual tissues in the formation of changes are shown in Table 1.

**Table 1:** Participation of individual tissues in the construction of structural changes

Location of the change	Tissues that contribute to structural changes		
	Osteodentin	Tubular dentin, dental pulp cells, cartilage–like cementum (CC)	Osteodentin, tubular dentin, dental pulp cells, cartilage–like cementum (CC)
Changes with dentin deformation		30	15
Changes within the pulp cavity	23	14	6
Changes within the whole tooth			2

**Fig. 5:**
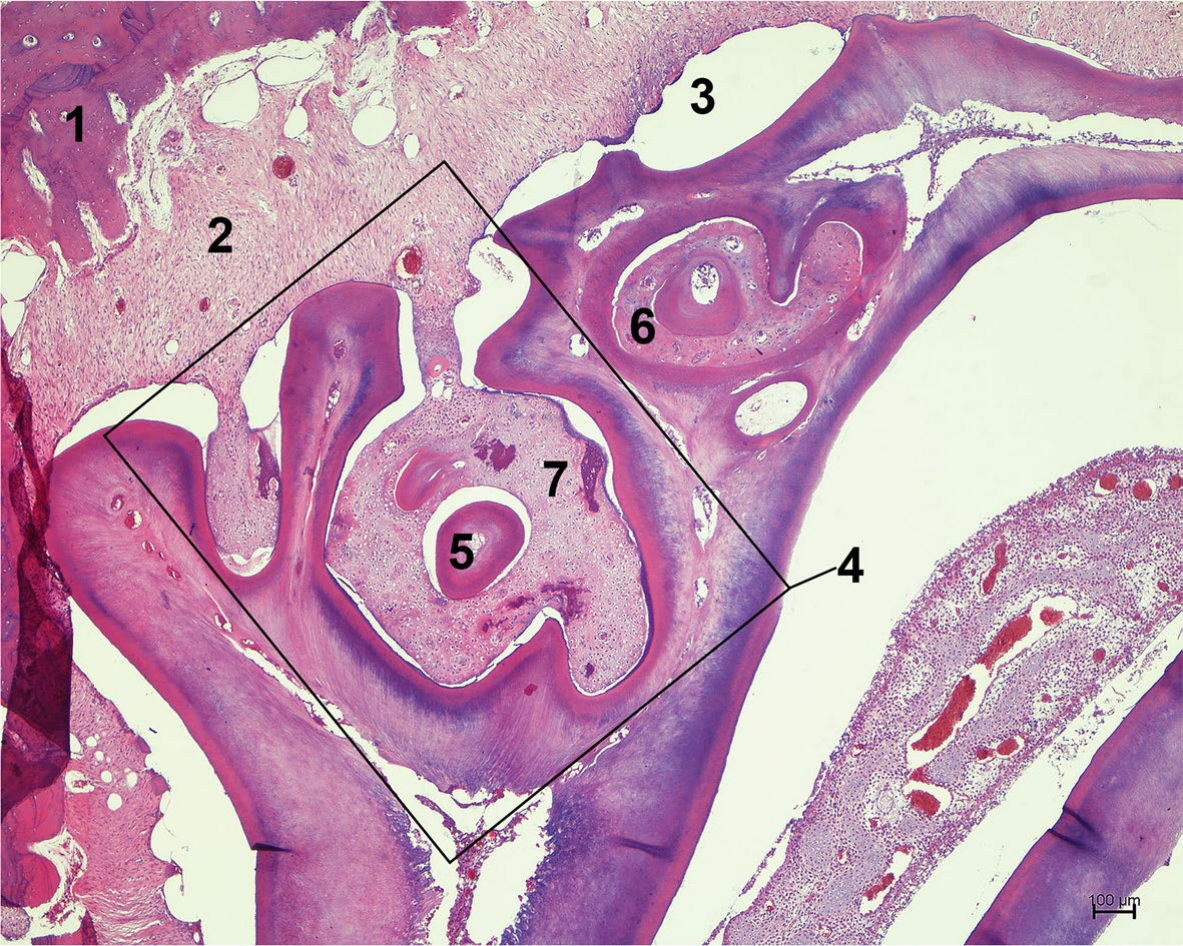
Transverse section of a guinea pig cheek tooth with structural alteration on the periphery of the tooth crown (H&E, × 40). 1. Alveolar bone, 2. periodontium, 3. enamel space, 4. protrusion of the dentin into the pulp cavity, 5. tubular dentin, 6. osteodentin, 7. cartilage-like cementum filling the indentation area

**Fig. 6:**
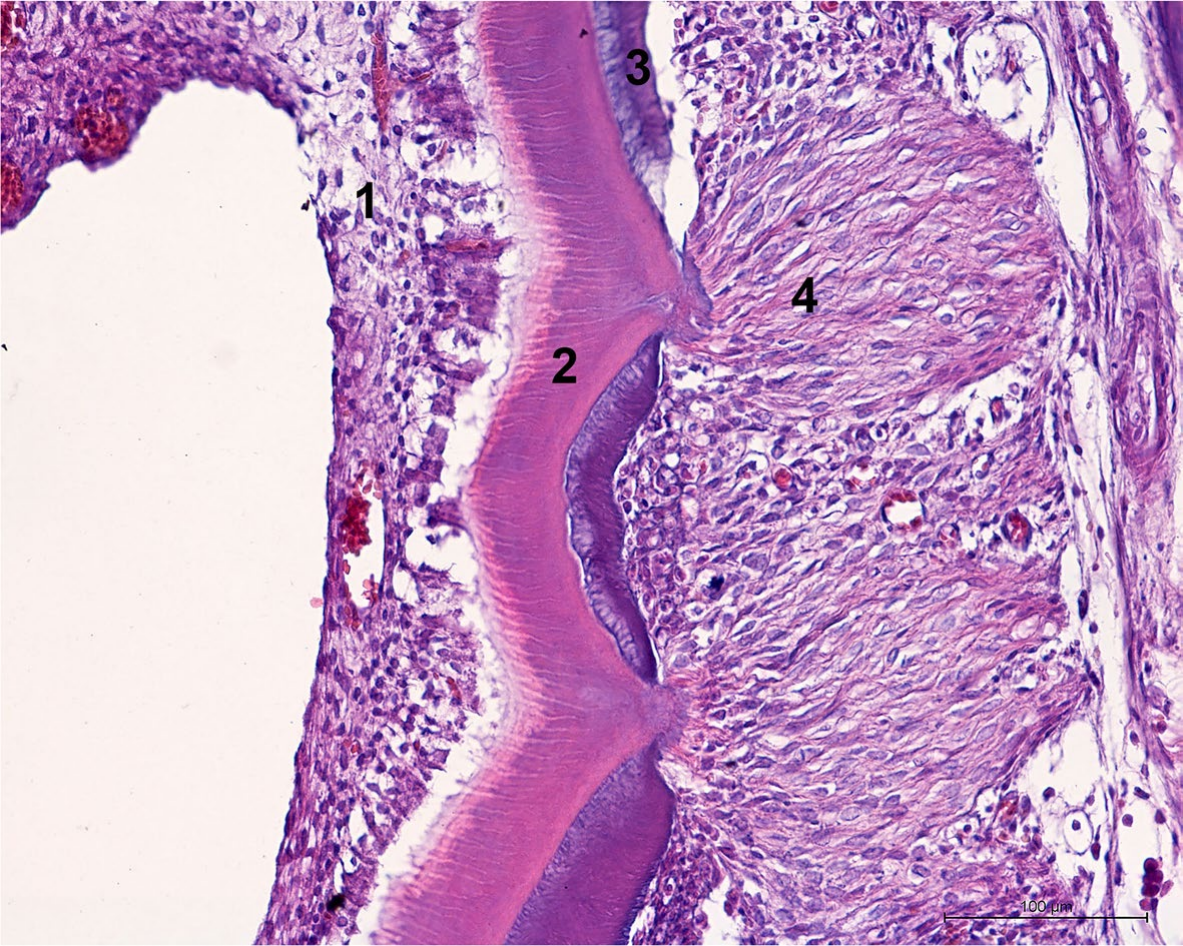
Transverse section of a guinea pig cheek tooth with structural alteration on the periphery of the tooth crown (H&E, × 200). The areas of dentin prominence are not covered with the enamel matrix, which disturbs the arrangement of the periodontal ligaments. 1. Germinal tissue, 2. dentin, 3. enamel matrix, 4. accumulated periodontal ligaments

**Fig. 7:**
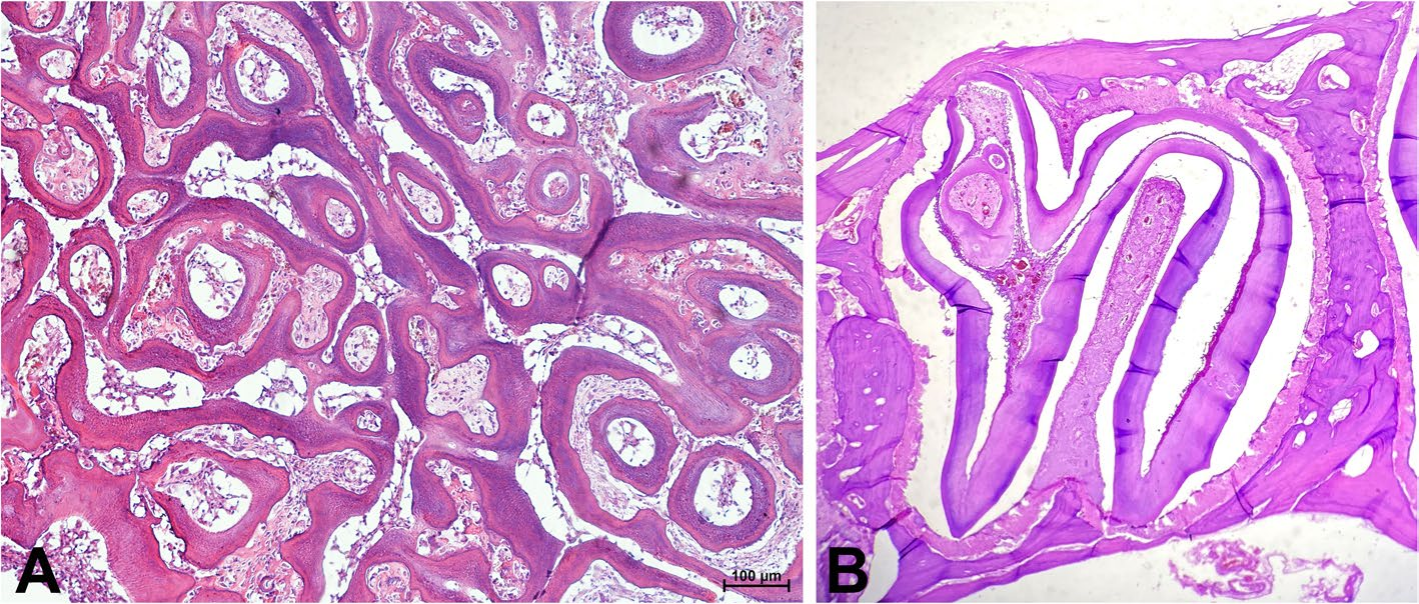
Transverse section of a cheek tooth with the change within the pulp cavity. 7a. Structural alteration built of tubular dentin that forms various shapes, the pulp cells are trapped between the bands and rings of the dentin (H&E, × 100). 7b. Two rings were built of tubular dentin in the central part of the pulp cavity. In the central part of the change, the osteodentin is trapped (H&E, × 10)

**Fig. 8:**
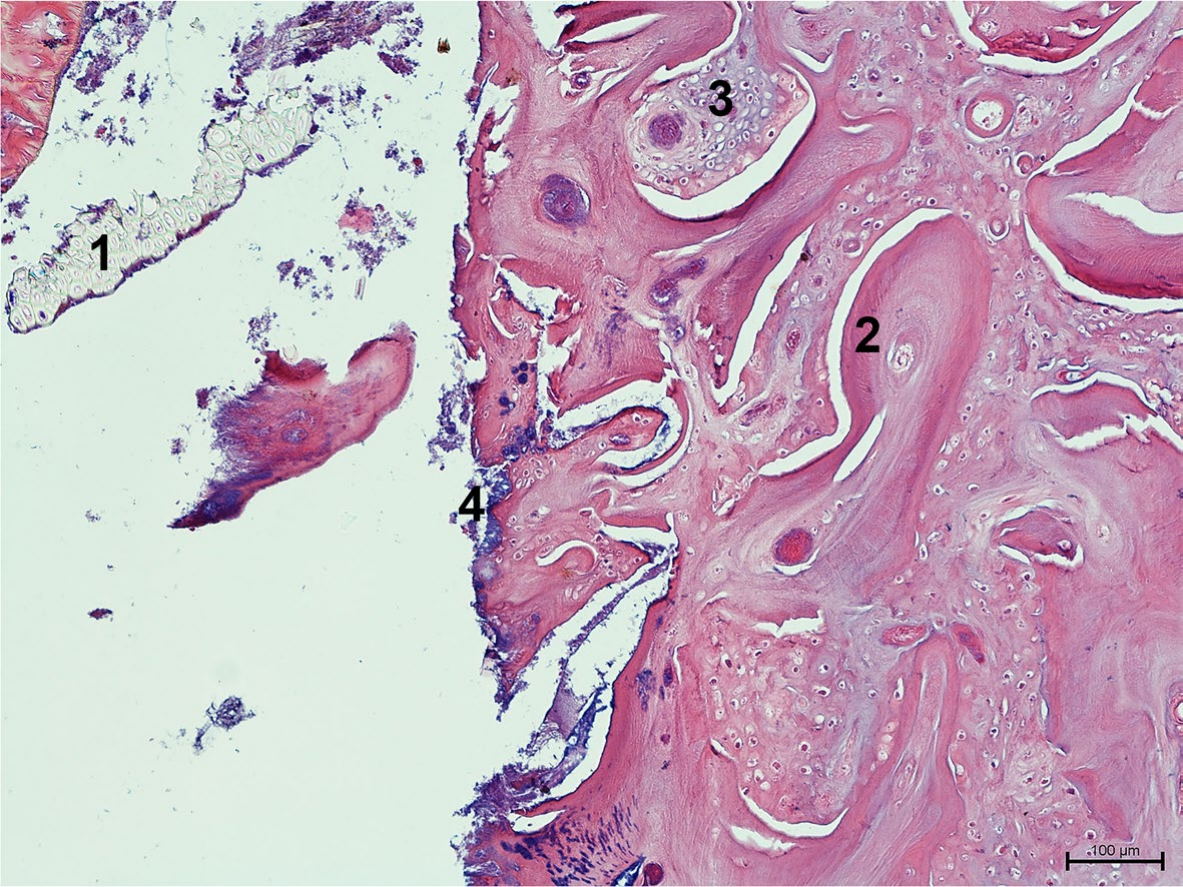
Longitudinal section of the clinical crown of a guinea pig cheek tooth. As a result of the loss of connection between the gingiva and structurally changed teeth, food debris penetrates the space between the two adjacent teeth. Bacteria present on the plant fragments penetrate deep into the tooth structure. Dentin, osteodentin and cartilage-like cementum breaks loose on the occlusal surface (H&E, × 100). 1. Plant material, 2. tubular dentin, 3. osteodentin, 4. bacteria

Alterations in dentin deformation are those in which the concavities observed above the apical bud towards the pulp cavity without breaking the dentin continuity or prominence (protrusions) resemble dentin fractures towards the periodontal space and alveolar bone. The space formed by the dentin indentation is filled with tissue of epithelial origin from which cementoblasts are formed and attached to the periodontium. In some cases, the presence of odontoblasts forming additional dentin bundles in the periodontal space was found (Fig. 5). Enamel participates in the formation of alterations in dentin deformation. However, in areas where the dentin protrudes towards the periodontal space, the enamel thins or disappears, which also affects the arrangements of periodontal ligaments (Fig. 6). The periodontal ligaments accumulate and concentrate on the dentin fragment that is lacking enamel (Fig. 6). No enamel was detected in the structural changes in the pulp cavity. In 23 of those changes, the presence of variously shaped formations composed of osteodentin was observed, which occurred individually to form a column extending vertically within the crown from the apex to the occlusal surface or in clusters of several smaller changes. The remaining 20 alterations in the structure were observed in variously shaped formations composed of tubular dentin. In this case, the arrangement of tubules was often disorganized (Fig. 7a). Dental pulp cells, cementoblasts, cementum and, in 6 cases, the osteodentin were trapped between dentin bundles or in rings made of tubular dentin (Fig. 7b). Regardless of the location of structural remodelling, the shape of the tooth and its size were changed, and the alveolar bone was remodelled by adjusting to the new shape of the tooth. In 29 cases, it was possible to evaluate the occlusal surface of the tooth crown, and in 8 of those cases, loss of the enamel, dentin and cementum were found on the edge of the altered part of the tooth. In such cases, the presence of plant remains and bacteria was observed in the interdental space and within the tooth tissues on the occlusal surface (Fig. 8).

## Discussion

A macrodont is by definition a tooth that is larger than normal [12]. This statement is also found in specialized literature, both veterinary and dental [7, 8, 10, 12–14]. In the current study, the presence of characteristic structural changes was found within the macrodontic cheek teeth of the guinea pig. Similarly, one author observed structural deformities and splitting of the teeth with enlarged outlines [7]. The same author used the terms “giant tooth” and “crippled teeth” to describe this pathology. It is difficult to compare the observed structural alterations to possible changes found in human macrodontic teeth, as there are few papers in the available literature describing the histopathology of these teeth. However, according to Komatsu et al*.,* the development of macrodontic teeth in humans occurs in the bell stage. The constantly growing cheek tooth of the guinea pig remains in the bell stage throughout the animal’s life. In the work of Komatsu et al., histological examination of the human macrodontic tooth showed enamel hypoplasia, dentin abnormalities, hypertrophy of the cementum and immature enamel. Similar changes were observed in the examined cheek teeth of the guinea pig in areas where there was thinning or complete loss of the enamel on the dentin surface. In such situations, the cementum filled the areas where dentin had been driven into the pulp cavity. Köstlinger et al*.* [8, 9] found that 90% of macrodontic cheek teeth are M2 and M3. We obtained similar results during histological evaluation, as 87% of lesions in our study were located within M2 and M3 teeth.

Due to the presence of structural alterations in the examined teeth, the possibility of odontogenic tumours was suspected. Structural changes in macrodontic teeth of guinea pigs are composed of physiologically occurring hard and soft tissue of the tooth with disordered arrangement, whereas the cells do not show any signs of atypia. The possibility of odontogenic cysts was rejected first. Changes of epithelial origin not containing ectomesenchymal components were also eliminated (ameloblastoma). Alterations containing both epithelial and ectomesenchymal components in the structure (ameloblastic fibroma, ameloblastic odontoma, dentinoma, odontoma) were carefully analysed [15]. Among many odontogenic tumours, the posted description is most compatible with an odontoma. Odontomas are benign tumourlike malformations of the hard tissue of the tooth and arise from disorders of the division of the dental lamina. They are built of epithelial and ectomesenchymal components; hence, in the structure, we can see tissues such as enamel, dentin, cementum, and dental pulp, and their arrangement can be normal or disrupted, but the cells do not show changes. Due to their structure, odontomas are more likely to be classified as hamartomas (nonneoplastic tumours). There are 2 types of odontomas: compound odontoma (*odontoma compositum*), in which altered tissues form structures that anatomically resemble teeth (odontoids), and small remnants of epithelial tissue are found within the changes. The second type is called complex odontoma (*odontoma completum*), which is a chaotic mass of hard and soft tissue of the tooth that shows no morphological resemblance to a properly formed tooth [13, 16, 17]. Variations of this type have thus far been described for many animal species, including dogs, cats, voles, [18] squirrels [19], prairie dogs [20–22], mice [23, 24], rats [25], degus [26], and guinea pigs [27]. However, due to the different structures of brachydontic and elodontic rodent teeth, a different name has been proposed in the literature for the change histologically resembling the odontoma of anelodontic teeth. Boy and Steenkamp proposed the term “elodontoma” for a change diagnosed in a squirrel. In contrast, Pelizzone et al. suggested the term “pseudo-odontoma” to describe a change within the incisor of a prairie dog.

From the work of Capello et al*.,* we only know that the changes diagnosed in 2 guinea pigs showed features of elodontoma and were haphazardly composed of hard and soft tissues of the tooth. We found similar structural changes in histologically examined guinea pig teeth; however, in our case, the alterations were localized within the pulp cavity or at the periphery of the tooth crown, and in the 2 cases described by Capello et al*.,* the changes were localized outside the apex and showed local expansion.

Boy and Steenkamp also described complex odontoma type changes found in squirrels. In such a case, similar to guinea pig [27], the presence of changes composed of hard and soft tissues of the tooth with disordered arrangement located outside the apex was described. Changes in the borders of the tooth crown without the formation of a periapical odontogenic mass were also described. In such cases, deformation of the apex and corrugated enamel were found. Similar changes were noted during histologic evaluation of guinea pig macrodontic teeth in the work conducted (Fig. 4). Moreover, Boy and Steenkamp observed that most of the changes similar to the teeth we examined spread from the apex to the occlusal plane.

**Fig. 9:**
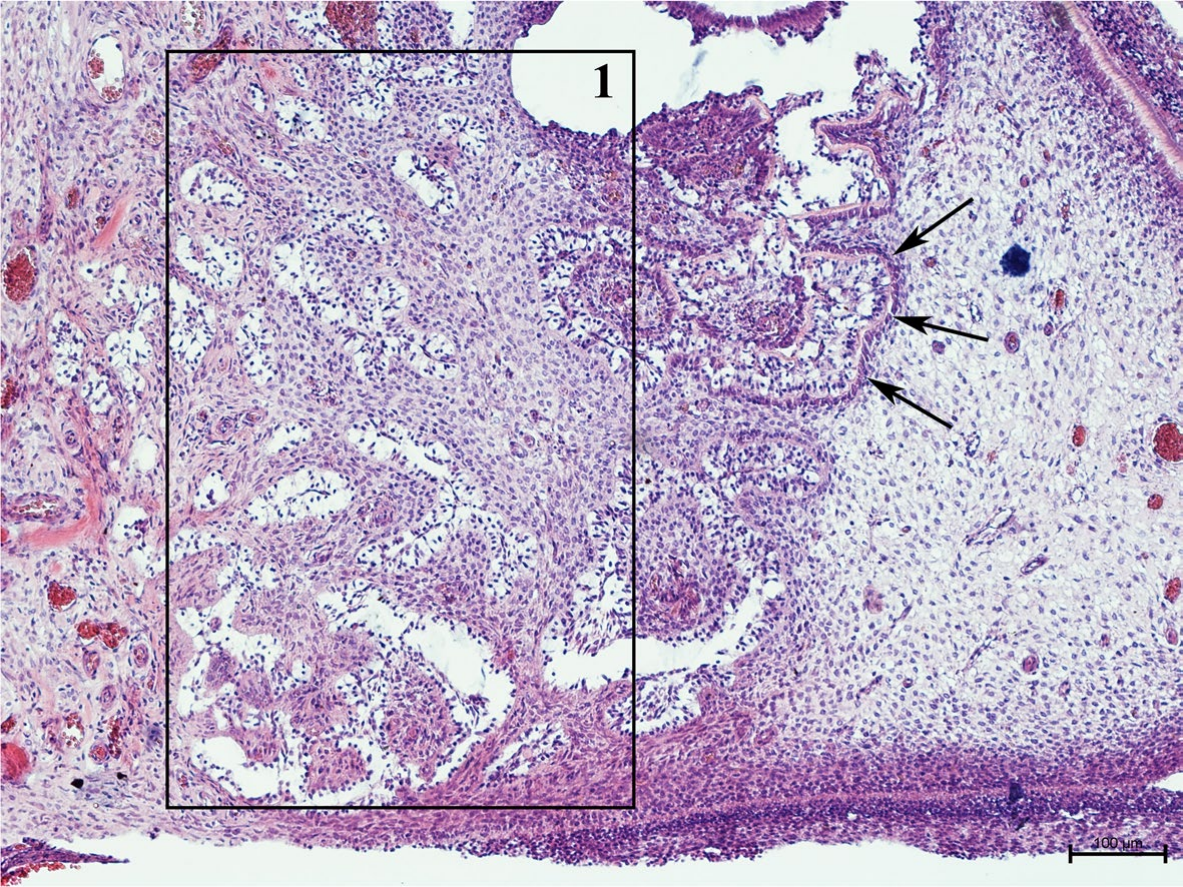
Transverse section by the apex of the guinea pig cheek tooth with structural alteration within the pulp cavity. 1. Within the pulp cavity, haphazard clusters of the epithelial tissue surrounded by the pulp cells are visible. Arrows indicate the bounds of the dentin (H&E, × 100)

Histological description of complex odontoma found in prairie dogs largely resembles changes found in guinea pig macrodontic teeth. In both cases, islands of epithelial and mesenchymal tissue forming tooth-like structures were found. Such changes in guinea pig teeth are shown in Fig. 9. The alterations were built of predentin, dentin and cementum, and the arrangement of these tissues was disordered, similar to the macrodontia of guinea pigs [20].

Dayan et al*.* described a case of complex odontoma in mice. Histological examination revealed the alteration built, as in previous cases, of enamel, dentin, cementum and connective tissue. Hard tissues formed island-like structures and were mixed with surrounding connective tissue. This image resembles structural changes in guinea pig macrodontic teeth (Fig. 7a). However, for the second time [23, 26], the presence of a fibrous capsule surrounding the change from the outside and beyond the apex was observed. This type of change was not found in the evaluation of guinea pig macrodontic teeth.

An interesting description of the complex odontoma was made by the authors of works conducted on *op/op* mice [24], *op/op* rats [25], and *ia* rats [28] in which the development of this pathology was described as a result of osteopetrosis. Osteopetrosis is an inherited metabolic bone disease in which the process of bone resorption is disrupted. Accumulating bone trabeculae invade the enamel organ through which separate fragments of daughter tooth germs capable of further production of the tooth tissue arise. During the evaluation of macrodontic teeth of guinea pigs, no expansion of bone tissue towards the apex was observed, but detached fragments of the apical bud or cracks in the dentin layer and inflow of pulp cells into the periodontal space or from the periodontal space towards the tooth pulp were observed. The structural alterations described in mice and rats also show considerable similarity to those observed in our study. In the studies by Ida-Yonemochi et al*.*, Philippart et al*.* and Schour et al*.* and in our study, disorganized cells of epithelial and mesenchymal origin formed various structures and rings. Trapped pulp cells were found between wavy bands and in the centre of the rings. Moreover, osteodentin was found in the formation of structural changes in rats, as well as in macrodontic teeth of guinea pigs. Osteodentin is mineralized tissue physiologically found in the incisors of rats [29–31] and the incisors and cheek teeth of guinea pigs [32]. It is a tissue of mesenchymal origin resembling bone with lacunes in which single cells are trapped. The osteodentin is surrounded by tubular dentin all around. It was observed in the top of the pulp cavity. It is suspected that its function is to seal the pulp cavity on the occlusal surface to protect the pulp cavity from the influx of pathogens from the oral cavity [30, 32]. The presence of this tissue in healthy rat teeth explains its occurrence in the structure of complex odontomas since it is a change built of physiologically existing tissues with a disordered arrangement. This observation does not yet conclusively establish that the structural changes in macrodontic teeth of the guinea pig qualify as odontomas but suggests that osteodentin should be present in the structure of the odontoma in the guinea pig. The papers cited in this paragraph found no connective tissue capsule surrounding the odontoma from the outside.

The presence of a ring-shaped deformity with centrally trapped pulp cells and peripherally located odontoblasts also resembles the description of complex odontoma in voles [18]. In this rodent, the occurrence of osteodentin in the odontoma structure was also found, and the presence of a fibrous capsule was not described. However, the changes in the maxillary incisors showed expansion towards the surrounding bone. In the same work, a description of complex odontoma in a dog and cat was also compiled. Similar to structural alterations in macrodontic cheek teeth of a guinea pig, the presence of tubular and atubular dentin of various shapes, enamel dysplasia and trapped pulp cells were shown.

## Conclusion

The presence of structural alterations in teeth with increased size (macrodonts) has already been reported in the literature. The collected data allow us to conclude that the macrodontic tooth of a guinea pig is not only a tooth with an increased outline that is larger than the others but also one that has characteristic structural changes. The cells of epithelial and mesenchymal origin within the altered teeth do not show features of atypia and thus indicate that the structural changes do not show a neoplastic origin. Among many described odontogenic tumours, the structural alterations of macrodontic teeth most strongly resemble those described for complex odontoma. However, there are noticeable differences described by other authors, primarily the expansion of the changes beyond the apex or the presence of a connective tissue capsule at the periphery of the change. The alterations described in our study localized within the pulp cavity of the tooth or at the periphery of the crown, and the alveolar bone was remodelled to adapt to the new shape of the tooth. It seems that the term hamartoma is the most universal to describe the changes observed in macrodont, as it refers to a malformation made of atypically arranged, physiologically present tissues. However, a certain determination of what the structural changes are in macrodontic teeth of guinea pigs requires further study. From the data collected, it also appears that this is the first paper describing the histological structure of macrodontic teeth of guinea pigs.

## Data Availability

The data are not publicly available because they are histological slides, but we can share more images after contact with the corresponding author: Justyna Ignaszak–Dziech, justyna.dziech@gmail.com.

## References

[1] Dziech J, Piasecki T (2021). Anatomical and histological structure of the cavy’s teeth as a basis for diagnosis of dental diseases. Med Wet.

[2] Hunt AM (1959). A description of the molar teeth and investing tissues of normal guinea pig. J Dent Res.

[3] Jayawardena CK, Takahashi N, Takano Y (2002). A unique localization of mechanoreceptors in the periodontal tissue of guinea pig teeth. Arch Histol Cytol.

[4] Jayawardena CK, Takahashi N, Watanabae E, Takano Y (2002). On the origin of intrinsic matrix of acellular extrinsic fiber cementum: studies on growing cementum pearls of normal and bisphosphonate – affected guinea pig molars. Eur J Oral Sci.

[5] Moriyama K, Sahara N, Kageyama T, Misawa Y, Hosoya A, Ozawa H (2006). Scanning electron microscopy of the three different types of cementum in the molar teeth of the guinea pig. Arch Oral Biol.

[6] Wassermann F (1944). Analysis of the enamel formation in the continuously growing teeth of normal and vitamin C deficient guinea pigs. JDent Research.

[7] Böhmer E (2015). Dentistry in Rabbits and Rodents.

[8] Köstlinger S, Witt S, Fehr M (2021). Macrodontia in Guinea Pigs (Cavia porcellus) Radiological findings and localisation in 131 patients. J Exot Pet Med.

[9] Köstlinger S, Witt S, Fehr M. Radiological Appearance and Localization of Macrodontia in Guinea Pigs. In: Proceedings of the 3^rd^ International Conference on Avian, Herpetological and Exotic Mammal Medicine, Venice, March 27 to 29 2017. Venice; 2017. S. 614–5

[10] Schweda MC, Hassan J, Bohler A, Tichy A, Reiter AM, Kunzel F (2014). The role of computed tomography in the assessment of dental disease in 66 guinea pigs. Vete Record.

[11] Ali ZH, Mubarak R (2012). Histomorphological Study of Dentine Pulp Complex of Continuously Growing Teeth in the Rabbits. Life Sci J.

[12] Rohilla M (2017). Etiology of Various Dental Developmental Anomalies -Review of Literature. J Dent Probl Solut.

[13] Yoda T, Ishii Y, Honma Y, Sakai E, Enomoto S (1998). Multiple macrodonts with odontoma in a mother and son—a variant of Ekman-Westborg-Julin syndrome: Report of a case. Oral Surg Oral Med Oral Pathol Oral Radiol Endod.

[14] Komatsu T, Kurihara T, Ito Y, Lee MC, Miyagi A, Ikeda M (2012). Oral characteristics of a patient with Ekman-Westborg-Julin trait: a case history. Spec Care Dentist.

[15] Munday JS, Löhr ChV, Kiupel M. Tumors of the Alimentary Tract. In: Meuten D.J. Tumors in Domestic Animals, Fifth Edition. USA: John Wiley & Sons; 2017. p.

[16] Rejendra Santosh AB (2020). OgleOE. Odontogenic Tumors Dent ClinNorth Am.

[17] Studziński M, Jędrusik – Pawłowska M, Mazur M, Samad RA (2014). Odontoma – an odontogenic tumour. Case description and review of the literature. Mag Stom.

[18] Walsh KM, Denholm LJ, Cooper BJ (1987). Epithelial odontogenic tumours in domestic animals. J Comp Pathol.

[19] Boy SC, Steenkamp G (2006). Odontoma-like Tumours of Squirrel Elodont Incisors—Elodontomas. J Comp Pathol.

[20] Pelizzone I, Di Ianni F, Volta A, Gnudi G, Manfredi S, Bertocchi M (2017). Computed Tomographic of Incisor Pseudo – Odontomas in Prairie (Cynomys Ludovicianus). Vet Radiol Ultrasound.

[21] Phalen DN, Antinoff N, Fricke ME (2000). Obstructive Respiratory Disease in Prairie Dogs with Odontomas. Vet Clin North Am Exot Anim Pract.

[22] Wagner RA, Garman RH, Collins BM (1999). Diagnosing Odontomas in Prairie dogs. Exot DVM.

[23] Dayan D, Waner T, Harmelin A, Nyska A (1994). Bilateral complex odontoma in a Swiss (CD-1) male mouse. Lab Anim.

[24] Ida-Yonemochi H, Noda T, Shimokawa H, Saku T (2002). Disturbed tooth eruption in osteopetrotic (op/op) mice: histopathogenesis of tooth malformation and odontomas. J Oral Pathol Med.

[25] Philippart C, Arys A, Dourov N (1994). Experimental odontomas in osteopetrotic op/op rats. J Oral Pathol Med.

[26] Jekl V, Hauptman K, Skoric M, Jeklova E, Fictum P, Knotek Z (2008). Elodontoma in a Degu (Octodon degus). J Exot Pet Med.

[27] Capello V, Lennox A, Ghisleni G (2015). Elodontoma in Two Guinea Pigs. J Vet Dent.

[28] Schour I, Bhaskar SN, Greep RO, Weinmann JP (1949). Odontome-like formations in a mutant strain of rats. Am J Anat.

[29] Hosoya A, Nakamura H, Akahane S, Yoshiba K, Yoshiba N, Ninomiya T (2006). Immunohistochemical Study of Osteodentin in the Unerupted Rat Incisor. J Oral Biosc.

[30] Karim AC, Eddy EL (1984). A light and electron microscopic study of osteodentin formation in the rat incisor after adriamycin administration. Am J Anat.

[31] Takuma S, Yanagisawa T, Lin WL (1977). Ultrastructural and microanalytical aspects of developing osteodentin in rat incisors. Calcif Tissue Res.

[32] Holmstedt JO, McClugage SG, Clark JS, Guevara MJ (1977). Osteodentin Formation in Continuously Erupting Teeth of Guinea Pigs. J Dent Res.

